# An extraction-free and one-pot two-temperature CRISPR/Cas12b system for visual detection of Group B *Streptococcus* by integrating with RPA

**DOI:** 10.1128/jcm.00819-25

**Published:** 2025-09-19

**Authors:** Chengman Zhao, Ge Li, Chen Shen, Yanjun Xie, Yun Chen, Xiaobo Ying, Yan Chen, Chuanling Zhang

**Affiliations:** 1 Department of Internal Medicine, Affiliated Xiaoshan Hospital, Hangzhou Normal University 26494, Hangzhou, China; 2 Department of Internal Medicine, Hangzhou Mingzhou Brain Rehabilitation Hospital, Hangzhou, China; 3 Department of Clinical Laboratory, Affiliated Xiaoshan Hospital, Hangzhou Normal University 26494, Hangzhou, China; 4 Department of Obstetrics and Gynecology, Affiliated Xiaoshan Hospital, Hangzhou Normal University 26494, Hangzhou, China; 5 Translational Medicine Laboratory, Affiliated Xiaoshan Hospital, Hangzhou Normal University 26494, Hangzhou, China; Mayo Clinic, Baltimore, Maryland, USA

**Keywords:** Group B *Streptococcus*, CRISPR/Cas12b, LAMP, RPA, one-pot two-temperature, extraction-free

## Abstract

Group B *Streptococcus* (GBS) is a major cause of neonatal infections, and rapid detection is essential for timely clinical intervention. In this study, we developed an extraction-free, one-pot CRISPR/Cas12b assay for visual detection of GBS by combining with isothermal amplification, including loop-mediated isothermal amplification (LAMP) and recombinase polymerase amplification (RPA). The results showed that LAMP-CRISPR/Cas12b outperformed RPA-CRISPR/Cas12b system across all template concentrations, especially in low-copy template (30 and 10 copies/test) detection. To enhance the detection performance of RPA-CRISPR/Cas12b, we introduced a two-temperature protocol, with RPA reaction at 39°C followed by Cas12b activation at 62°C. Through the two-temperature approach, the detection rate of RPA-CRISPR/Cas12b system was significantly improved even in low-copy samples, achieving a sensitivity of 10 copies/test (1 copy/μL). Clinical validation using 60 vaginal-rectal swab samples showed 96.7% and 98.3% of concordance when compared to culture and qPCR methods, respectively. This assay offers a rapid (<1 h), highly sensitive, and user-friendly solution without requiring nucleic acid extraction or sophisticated instruments. Its compatibility with visual signal detection makes it ideal for point-of-care testing, especially in low-resource or time-sensitive settings. The platform can be adapted for broader pathogen detection in future field diagnostics.

**IMPORTANCE:**

This study presents a rapid, convenient, and highly accurate method for Group B *Streptococcus* (GBS) detection by integrating the CRISPR/Cas12b system with recombinase polymerase amplification, an isothermal nucleic acid amplification technique. To streamline the workflow, we established a one-pot, extraction-free assay that significantly reduces the detection time. Through the systematic optimization of the dual-temperature conditions, we enhanced the amplification efficiency of target DNA, thereby improving the sensitivity of the CRISPR/Cas12b system. Additionally, the incorporation of a UV-visible detection system enables visual readout, facilitating instrument-free testing suitable for point-of-care (POC) applications.

## INTRODUCTION

Group B *Streptococcus* (GBS), or *Streptococcus agalactiae*, is a gram-positive bacterium that poses significant risks to neonates, pregnant women, and individuals with chronic conditions (1). As a leading etiological agent of neonatal sepsis, pneumonia, and meningitis, GBS is also linked to maternal infections such as urinary tract infections and endometritis (2, 3). Approximately 25% of healthy women are colonized with GBS in their gastrointestinal and genitourinary tracts, with vertical transmission to newborns primarily occurring during childbirth. The rapid progression and high mortality rate of neonatal GBS infections necessitate early detection and immediate intervention, particularly through intrapartum antibiotic prophylaxis (4, 5). While universal screening and antibiotic prophylaxis in high-income countries have dramatically reduced the incidence of early-onset GBS disease (6), low- and middle-income regions continue to face a substantial burden due to limited access to timely and accurate diagnostics (7, 8). This disparity underscores the critical need for rapid and accessible detection methods to enable early treatment and improve outcomes.

Conventional GBS diagnosis relies on bacterial culture, which, despite its reliability, is labor-intensive, time-consuming (requiring 24–48 h for results), and dependent on specialized laboratory infrastructure (9, 10). Moreover, techniques such as polymerase chain reaction (PCR) provide faster and more sensitive alternatives by enabling direct pathogen detection from clinical samples (4, 11, 12). However, the high cost and technical complexity of PCR-based methods often restrict their utility in resource-limited settings.

To address these limitations, isothermal amplification methods including loop-mediated isothermal amplification (LAMP) and recombinase polymerase amplification (RPA) have emerged as promising alternatives. LAMP is a rapid, highly sensitive nucleic acid amplification technique that operates at a constant temperature (60–65°C), eliminating the need for thermal cyclers (13, 14). Nevertheless, its requirement for multiple primers targeting distinct regions complicates assay design and increases susceptibility to non-specific amplification (15). In contrast, RPA functions at lower temperature (37–42°C) and employs only two primers, enabling faster setup and greater compatibility with temperature-sensitive systems such as those based on clustered regularly interspaced short palindromic repeats (CRISPR)-based detection (16, 17). Nonetheless, RPA is prone to background amplification and primer-dimer artifacts, particularly in complex sample matrices. Despite these challenges, both LAMP and RPA exhibit strong potential when integrated with CRISPR/Cas systems, where the sequence-specific cleavage activity of Cas proteins enhances assay specificity and minimizes false positives, making them ideal for rapid, point-of-care (POC) diagnostics (18, 19).

CRISPR/Cas systems, originally developed for genome editing, have been repurposed for highly specific nucleic acid detection (20). CRISPR-based diagnostics leverage the programmability and *trans*-cleavage activity of Cas enzymes to enable real-time signal generation, offering unparalleled specificity and sensitivity (21). Pioneering CRISPR diagnostic platforms, including SHERLOCK (Cas13a) (22), DETECTR (Cas12a) (23), and HOLMES (Cas12b) (24), have demonstrated rapid and accurate pathogen detection. However, CRISPR-based GBS detection remains underexplored, and existing assays often involve multi-step workflows requiring nucleic acid extraction, separate amplification, and post-amplification detection, limiting their practicality for field or POC use (25, 26). For instance, Jiang et al. (26) developed a two-pot RPA-Cas13 detection system, which necessitated T7 promoter-tagged primers and *in vitro* transcription, increasing complexity and cost. In a two-pot assay, both systems required manual liquid transfer, raising contamination risks.

One-pot CRISPR-based assay, which integrates amplification and detection in a single reaction, represents a major advancement in molecular diagnostics, simplifying workflows and reducing contamination (27). Strategies such as phase separation (28), suboptimal protospacer adjacent motif (PAM) sites utilization (29), Cas protein engineering (30), and chemically modified crRNA (31) have been explored to enable effective one-pot detection. In GBS diagnostics, one-pot assay has been achieved through crRNA modifications and suboptimal PAM selection (32). While valuable, these approaches introduce technical complexity, increase costs (33), and limit generalizability due to the non-universality of suboptimal PAM sequences.

To overcome these limitations, we developed a streamlined, universal one-pot GBS detection system leveraging the thermostable AapCas12b enzyme, which exhibits broad temperature adaptability (34). In this study, we first established parallel one-pot workflows using LAMP-CRISPR/Cas12b and RPA-CRISPR/Cas12b, finding that the RPA-based system exhibited lower sensitivity. To enhance performance, we implemented a one-pot two-temperature strategy, hypothesizing that temperature modulation could optimize the trans-cleavage activity of AapCas12b. By evaluating the *cis*-cleavage and *trans*-cleavage efficiency across a temperature gradient, we identified an optimal switching point, significantly improving the signal-to-noise ratio and low-template detection. Furthermore, our system enables instrument-free visual readout under UV light, simplifying interpretation. To maximize POC applicability, we integrate extraction-free sample processing with the one-pot two-temperature reaction and visual fluorescence detection. This system eliminates nucleic acid extraction, consolidates all steps into a single tube, and provides naked-eye result interpretation. Compared to conventional multi-step assays, crRNA modifications and suboptimal PAM-based methods, our system offers a cost-effective, equipment-free, and user-friendly solution for GBS detection (Table S5), making it suitable not only for clinical laboratories but also for decentralized settings where speed, simplicity, and minimal infrastructure are paramount.

## MATERIALS AND METHODS

### Workflow of the rapid, extraction-free one-pot two-temperature RPA-CRISPR/Cas12b system for visual detection of GBS

The procedure begins with minimal simple pretreatment, and DNA is released directly from swab samples by incubation in buffer at room temperature or via brief heat treatment (95°C for 5 min). The lysate is then added to a pre-mixed one-pot reaction containing all necessary RPA and CRISPR/Cas12b reagents.

Amplification is performed isothermally at 39°C for 40 min using RPA. Subsequently, the temperature is raised to 62°C for 5 min to activate AapCas12b-mediated *trans*-cleavage, generating a fluorescence signal detectable by the naked eye under blue or UV light. With a total assay time under 1 h, this system is particularly suitable for resource-limited settings or scenarios requiring rapid diagnostics, providing a sensitive, equipment-free visual readout.

### Primer and sgRNA design

To establish a highly sensitive and specific detection system for GBS, we identified a conserved region within the *cfb4* gene (GenBank accession no. HQ148672.1) through comprehensive multiple sequence alignment. This target sequence was subsequently cloned into the pUC57 plasmid to generate a recombinant plasmid, which functioned as a positive control (PC) throughout our experiments. For nucleic acid amplification, we designed 10 primer sets for both LAMP and RPA assays. LAMP primers were designed using the NEB LAMP Primer Design Tool (https://lamp.neb.com/#!/), while RPA primers were optimized using SnapGene software (V.6.0.2). Detailed primer sequences are provided in Table S1 (LAMP) and Table S2 (RPA).

To evaluate LAMP amplification efficiency, we incorporated Syto9/SYBR Green into the reaction mixture for real-time fluorescence monitoring, using no-template control (NTC) as a negative reference. Amplification efficiency was determined based on the time-to-positive (TTP) signal: shorter TTP values indicate higher primer efficiency.

For RPA primer pairs, amplification efficiency was evaluated by analyzing the products via 1% agarose gel electrophoresis. A higher amplification efficiency corresponds to greater product yield, which is reflected by a stronger intensity of the electrophoretic band.

To enable CRISPR-based detection, we designed multiple single-guide RNAs (sgRNAs) targeting distinct regions of the *cfb4* gene. The sgRNA sequences are listed in Table S3. All oligonucleotides, including primers and sgRNA reverse templates, were synthesized by Sangon Biotech (Shanghai, China). For sgRNA preparation, we employed the Cas12b High Yield sgRNA Synthesis and Purification Kit (Tolo Biotech, China; Cat. No. 31904), strictly adhering to the manufacturer’s protocol to ensure high-quality sgRNA production.

### AapCas12b and LAMP-mediated one-pot testing

The integrated LAMP-CRISPR/AapCas12b detection system was performed in a 25 µL reaction volume containing:

1. 2.5 µL 10 × LAMP buffer (Tolo Biotech, Cat. No. 25102);
2. 1.0 µL dNTP mix (25 mM; Hongene, Cat. No. DN32);
3. 2.0 µL MgSO₄ (100 mM; NEB, Cat. No. B1003S);
4. 2.5 µL 10 × LAMP primer mix;
5. 6.0 µL glycine (2 M; Sangon Biotech, Cat. No. A610235);
6. 1.0 µL Bst DNA polymerase (Tolo Biotech, Cat. No. 25102);
7. 0.125 µL AapCas12b (10 µM; Tolo Biotech, Cat. No. 32118);
8. 0.5 µL sgRNA (10 µM);
9. 1.25 µL ROX-labeled ssDNA reporter (10 µM; Sangon Biotech);
10. 6.125 µL nuclease-free water (Solarbio, Cat. No. R1600); and
11. 2.0 µL DNA template.

The reaction was incubated at 60°C for 45 min with real-time fluorescence monitoring (at 30-s intervals) to simultaneously track both LAMP amplification and Cas12b-mediated cleavage activity. A 200 µL transparent flat-cap PCR tube was used for the assay, with the hot lid function enabled to prevent evaporation and contamination. Real-time fluorescence measurements were performed on a SLAN-96P real-time fluorescence quantitative PCR analyzer (Hongshi, China), with signals acquired every 30 s to simultaneously monitor LAMP amplification and Cas12b-mediated trans-cleavage activity.

### AapCas12b and RPA-mediated one-pot testing

The integrated one-pot RPA-CRISPR/AapCas12b detection was performed in a 25 µL reaction system containing:

1. 12.5 µL Buffer A (lyophilized RPA reagents; ZHONGCE Biotech);
2. 1.25 µL Forward primer (10 µM);
3. 1.25 µL Reverse primer (10 µM);
4. 1.25 µL Buffer B;
5. 0.125 µL AapCas12b (10 µM);
6. 0.5 µL sgRNA (10 µM);
7. 1.25 µL ssDNA reporter (10 µM);
8. 2.5 µL 10 × HOLMES buffer;
9. 2.375 µL Nuclease-free water; and
10. 2.0 µL DNA template.

The reaction was incubated at 39°C for 45 min with real-time fluorescence measurement (at 30-s intervals).

### The sensitivity and specificity analysis on one-pot assays

The analytical sensitivity of the one-pot detection system was determined using serial dilutions (10–1,000 copies/test) of the *cfb4*-containing recombinant plasmid. Eight replicates were set in the high-concentration template groups (1,000 and 300 copies/test), and 16 replicates were performed in the medium-to-low concentration (100, 30, and 10 copies/test) groups. Fluorescence signals from both target amplification and CRISPR-mediated detection were analyzed.

The assay’s specificity was validated against a panel of 11 clinically relevant non-target bacterial strains: *Candida albicans* (Robin) Berkhout DNA standard, *Mycoplasma genitalium* DNA standard, *Neisseria gonorrhoeae* ATCC 49226, *Pseudomonas aeruginosa* NGATCC 27583, *Enterobacter hormaechei* ATCC 700323, *Escherichia coli* ATCC 8739, *Staphylococcus saprophyticus* ATCC BAA-750, *Enterococcus casseliflavus* ATCC 700327, *Staphylococcus aureus* ATCC 25923, *Streptococcus pneumoniae* ATCC 49619, and *Staphylococcus epidermidis* ATCC 12228.

The *cfb4*-containing recombinant plasmid was used as the PC, while nuclease-free water served as the NTC. All reactions were performed in triplicate to ensure reproducibility.

### *Cis*-cleavage and *trans*-cleavage assay

To characterize the temperature-influenced cleavage activity of Cas12b, we performed systematic enzymatic assays across a broad temperature gradient (37°C, 39°C, 42°C, 45°C, 48°C, 55°C, 60°C, 62°C, 65°C, and 68°C) to identify optimal reaction conditions. For fluorescence-based characterization of both *cis*-cleavage and *trans*-cleavage activity, the reaction system contained two parallel reporter constructs: (i) a 5 µM FAM-BHQ1-labeled double-stranded DNA (dsDNA) substrate (1:1 ratio) for monitoring *cis-*cleavage activity, and (ii) a 10 µM ROX-BHQ2-labeled single-stranded DNA (ssDNA) reporter for *trans*-cleavage detection. Following temperature-controlled incubation, real-time fluorescence measurements were acquired using a quantification PCR (qPCR) instrument (Hongshi, Shanghai, China). Complete probe sequences are detailed in Table S4.

### Optimized one-pot two-temperature RPA-CRISPR/Cas12b assay protocol

The detection was performed using a modified version of the standard RPA-CRISPR/AapCas12b one-pot assay with the following key adaptations:

1. Reaction volume optimization:
  1. Total volume reduced to 10 µL (from 25 µL); and
  2. Template input maintained at 10% vol (1 µL).
2. Temperature-phased reaction conditions:
  1. Amplification phase: 39°C for 40 min (RPA optimal conditions); and
  2. Detection phase: 62°C for 5 min (AapCas12b optimal cleavage temperature).

Fluorescence signals were recorded every 30 s throughout the reaction using a real-time fluorescence reader.

### Clinical samples

A total of 60 vaginal-rectal swab samples were collected from Zhejiang Xiaoshan Hospital under an ethically approved protocol (approval no. K2022035). Specimens were obtained using Copan swabs (Copan Diagnostics, CA), which maintain stability at 2–30°C (across refrigeration and room temperature) for both aerobic and anaerobic bacteria. Sample collection followed the 2020 American College of Obstetricians and Gynecologists guidelines for GBS screening (35). To optimize GBS detection, a single swab was used to sequentially collect vaginal secretions (lower vaginal introitus) and rectal samples (beyond the anal sphincter) without speculum assistance. All samples were free of blood contamination and particulate matter. Swabs were preserved in non-nutritive transport medium and processed within 24 h of collection. Following extraction-free pretreatment, samples were analyzed using our one-pot two-temperature RPA-CRISPR/Cas12b detection system.

### Culture method for the identification of GBS

Vaginal-rectal swabs were inoculated onto Stuart medium (Copan, Italy) at 37°C under 5% CO_2_ for 18–24 h, with negative cultures extended to 48 h (36). Characteristic β-hemolytic, gray-white colonies were sub-cultured and identified using the VITEK 2 automated system (bioMérieux) by clinical laboratory technicians at the Department of Clinical Laboratory, the Affiliated Xiaoshan Hospital, Hangzhou Normal University.

### qPCR assay

GBS detection was confirmed using the Diagnostic Kit for GBS DNA (PCR Fluorescence Probing) (Lot. No. 24006, Sansure Biotech, China) on a SLAN-96P fluorescent quantitative PCR system (Hongshi, China). The protocol included:

1. Uracil DNA glycosylase treatment (50°C, 2 min) for amplicon contamination control,
2. Taq enzyme activation (94°C, 5 min), and
3. 45 amplification cycles (94°C, 15 s, 57°C 30 s with FAM signal acquisition).

### Evaluation of extraction-free sample processing for one-pot detection

To assess the feasibility of simplified sample preparation, we performed parallel experiments using culture-confirmed GBS-positive vaginal-rectal swabs, comparing: (i) extraction-free methods with/without heat treatment, and (ii) extraction-free versus commercial kit-based nucleic acid isolation. Different kinds of Direct PCR Lysis Buffer purchased from Suzhou CretBiotech Ltd. (China) were used for comparison, including RLB15 (Cat. No. CNA02027), BL01 (Cat. No. CNA02033), and DirectStore (Cat. No. CSS03221). The core compositions of Direct PCR Lysis Buffer are Tris-HCl, KCl, and MgCl_2_, supplemented by Bovine Serum Albumin or gelatin. A commercial qPCR kit (National Medical Device Approval No. 20233401646, Sansure Biotech) was used for comparative analysis.

Three GBS-positive swabs were processed as follows:

Design 1: Heat Treatment Optimization

1. Swab heads were suspended in 600 µL physiological saline (10 s vortex);
2. 300 µL suspension was mixed with 300 µL 2× Buffer RLB15; and
3. The mixture was divided into two aliquots: (i) Untreated: Used directly for detection and (ii) Heat-treated: Incubated at 95°C for 5 min before testing.

Design 2: Extraction Method Comparison

Three additional positive swabs were processed using:

1. Standard protocol:
  - Saline suspension (600 µL, 10 s vortex);
  - Centrifugation (12,000 rpm, 2 min);
  - Supernatant removal (~100 µL residual); and
  - Magnetic bead-based extraction (Strong Fecal Genomic DNA Kit, Cat. No. CNA03290; elution in 80 µL).
2. Extraction-free protocol:
  - Pellet resuspension in 160 µL 1× Buffer RLB15; and
  - Division into two equal portions: (i) Direct testing; (ii) Heat treatment (95°C, 5 min) before detection.

### Statistical analysis

All statistical analyses were performed using GraphPad Prism software (version 10.2). The data were expressed as mean ± standard deviation (SD). Student’s *t*-test was employed for statistical comparisons, with significance levels defined as: *P < 0.05, **P < 0.01, ***P < 0.001, and ****P < 0.0001.

## RESULTS

### The establishment of one-pot LAMP-CRISPR/Cas12b and RPA-CRISPR/Cas12b systems

We designed 10 LAMP primer sets (Primers 1–10), with their product size ranging from 190 to 220 bp (Table S1). Primer amplification efficiency was evaluated using SYBR Green-based fluorescence analysis. According to the time needed for signal generation, LAMP Primers 1 and 10 showed superior amplification kinetics, as evidenced by their shorter TTP signals (Fig. S1A). However, Primer 1 exhibited non-specific amplification in NTC, prompting the selection of Primer 10 for all subsequent experiments.

For the RPA system, we designed three forward primers (F1–F3) and five reverse (R1–R5) primers. Agarose gel electrophoresis revealed that the F1/R1 primer pair generated the most intense amplification bands, followed by the F1/R2 combination (Fig. S1B), indicating superior amplification efficiency. Consequently, the F1/R1 primer pair was selected for all subsequent experiments.

**Fig 1:**
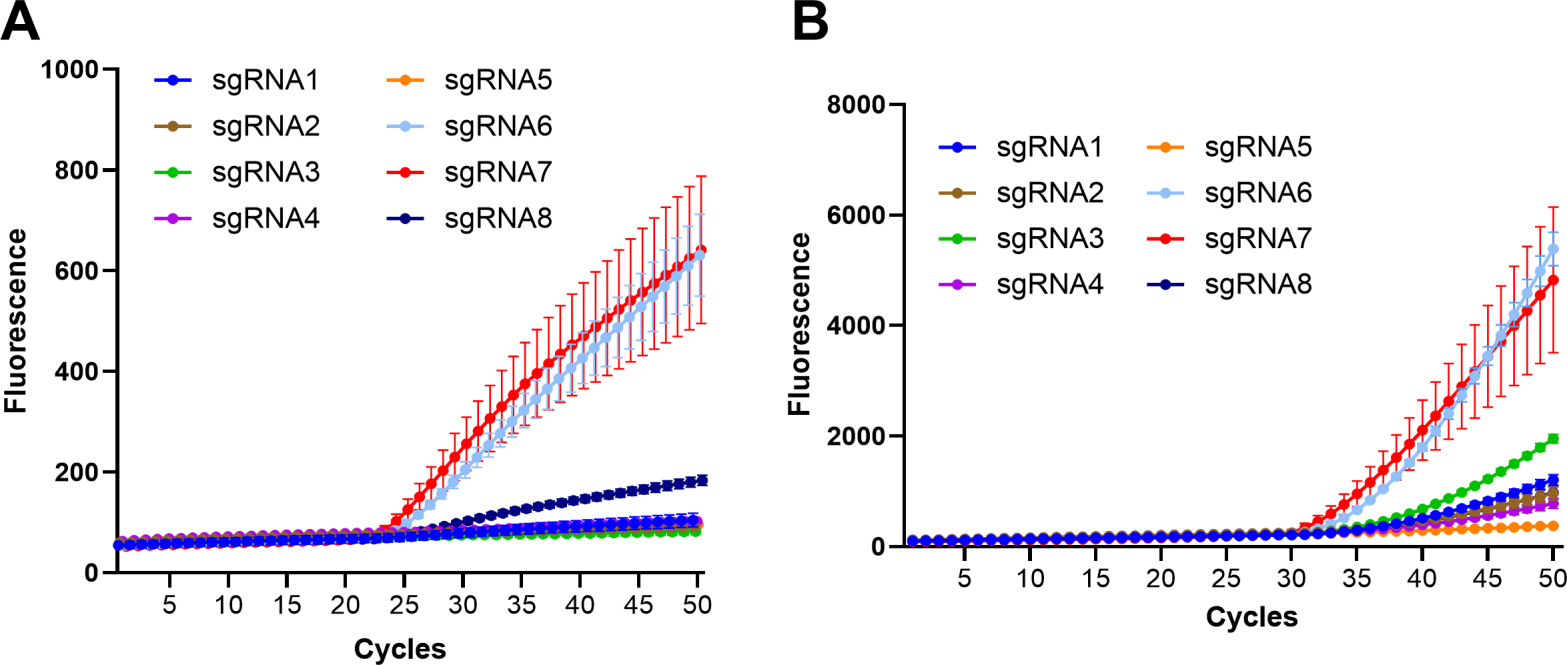
Screening of sgRNAs for LAMP- (**A**) and RPA-based (**B**) CRISPR/Cas12b detection systems. Seven sgRNAs (sgRNA1–7) targeting distinct regions of the *cfb4* gene were assessed. Both systems were tested with 10^5^ copies of the target template. LAMP reactions were carried out at 60°C for 45 min, while RPA reactions were conducted at 39°C for 45 min, with fluorescence measurements taken every 30 s.

The LAMP-CRISPR/Cas12b system exhibited optimal performance with sgRNA7, displaying the fastest and most robust fluorescence signal increase (Fig. 1A). Signal amplification initiated at approximately cycle 21, reaching peak intensity before cycle 25. sgRNA6 showed comparable but slightly reduced activity. In contrast, sgRNAs 1–5 and 8 demonstrated minimal to no fluorescence enhancement, indicating poor cleavage activity of Cas12b.

The RPA-CRISPR/Cas12b system showed distinct kinetic characteristics (Fig. 1B). While sgRNA6 and sgRNA7 again demonstrated the best performance, signal onset occurred later (cycles 30–35) compared to the LAMP-based system. Notably, despite this delayed initiation, the RPA system generated higher endpoint fluorescence intensity (above 4000 at cycle 50) than its LAMP counterpart (above 600 at cycle 50). These results suggest that while the LAMP-CRISPR/Cas12b system offers faster detection, the RPA-CRISPR/Cas12b system provides superior final signal strength.

### Sensitivity and specificity assessment of one-pot detection systems

**Fig 2:**
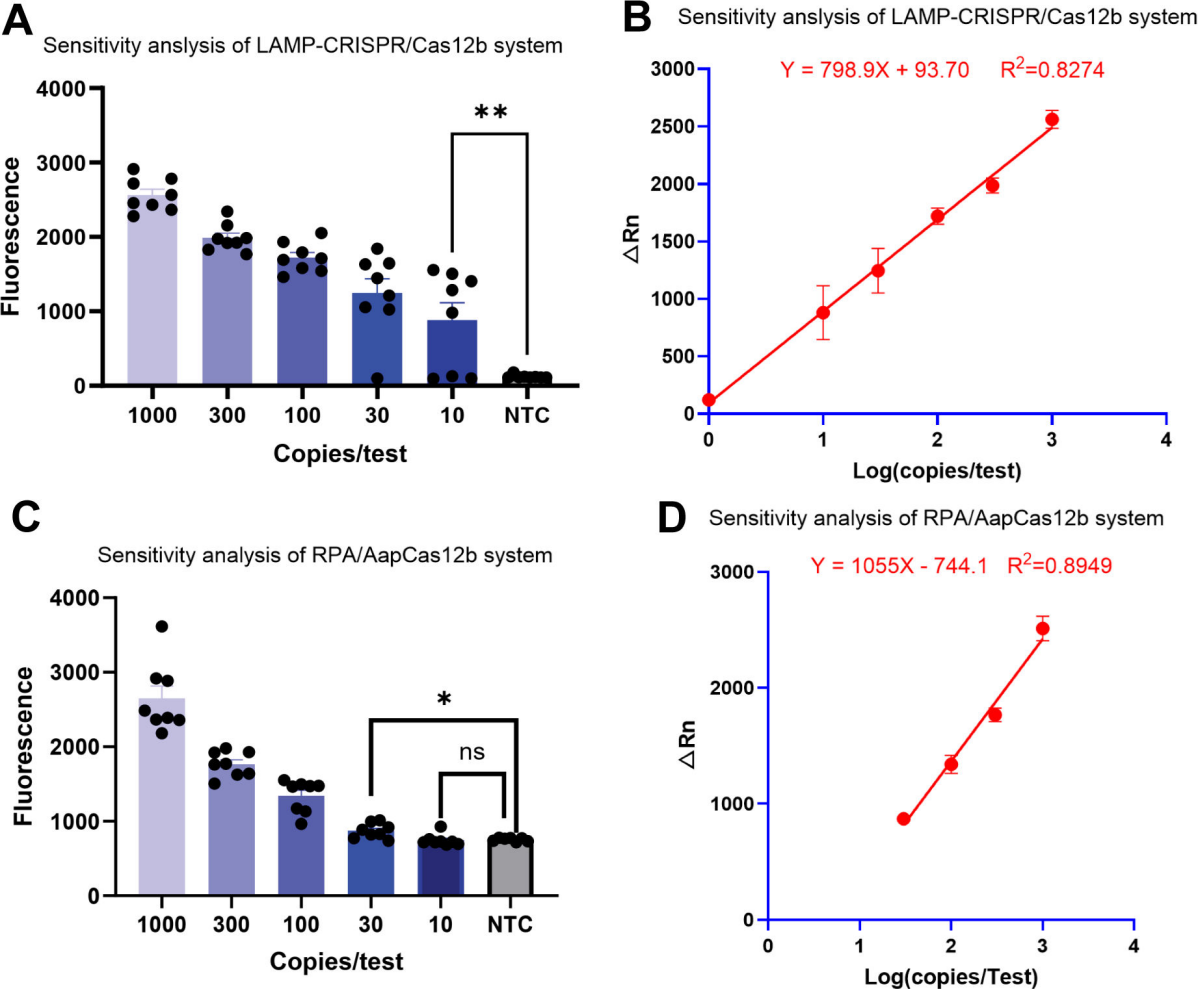
Sensitivity comparison of the one-pot LAMP-CRISPR /Cas12b (**A and B**) and RPA-CRISPR/Cas12b (**C and D**) detection systems. (**A and C**) Real-time fluorescence amplification curves of LAMP- and RPA-based one-pot assays at varying template concentrations (1,000, 300, 100, 30, and 10 copies/test) (*P < 0.05, **P < 0.01, ns: no significance). (**B and D**) The line of best fit between endpoint fluorescence intensity and Log(copies/test), with linear equation and *R*^2^ value indicated. Eight replicates were set at 1,000 and 300 copies/test, and 16 replicates were detected at 100, 30, and 10 copies/test. NTC, no-template control.

In performing the one-pot LAMP-CRISPR/Cas12b system, the endpoint fluorescence intensity is increased along with the template concentration elevated, and a statistically significant difference is noted between 10 copies and NTC (*P* = 0.006) (Fig. 2A), indicating its high sensitivity in detecting low-copy targets. Perfect detections (100% positive rate) were observed in all 1,000 (8/8), 300 (8/8), and 100 (16/16) copies/test. The positive rate was 87.5% (14/16) detection at 30 copies/test; and 62.5% (10/16) detection at 10 copies/test. A strong positive linear correlation (*R*^2^ = 0.8274) was observed between endpoint fluorescence intensity and logarithmic template concentration (Fig. 2B), confirming reliable quantitative detection across the tested range.

Similarly, Fig. 2C displayed the results of sensitivity analysis on the one-pot RPA-CRISPR/Cas12b system. With the template concentration increased, the endpoint fluorescence intensity was also raised. However, no statistical difference was observed between 10 copies and NTC (*P* = 0.766), indicating a low detection confidence at this concentration. Compared to the NTC group, 30 copies/test of template yielded a significantly higher fluorescence intensity (*P* = 0.01). Complete detections (100% positive rate) were reported in templates ≥ 100 copies/test; 50% (8/16) of positive rate at 30 copies/test; and 25% (4/16) of positive rate at 10 copies/test. Figure 2D demonstrated a strong positive linear correlation (*R*^2^ = 0.8949) between endpoint fluorescence intensity and logarithmic template concentration, proving the availability of the one-pot RPA-CRISPR/Cas12b system.

The detection rate for each system is summarized in Table 1, revealing that LAMP-CRISPR/Cas12b outperformed RPA-CRISPR/Cas12b across all template concentrations, especially for low-copy targets (30 and 10 copies/test).

**TABLE 1:** Detection rate of one-pot LAMP-CRISPR/Cas12b and RPA-CRISPR/Cas12b systems in different concentrations of template (copies/test)

CRISPR-Dx	1,000 (copies/test)	300 (copies/test)	100 (copies/test)	30 (copies/test)	10 (copies/test)
LAMP-CRISPR/Cas12b	8/8	8/8	16/16	14/16	10/16
RPA-CRISPR/Cas12b	8/8	8/8	16/16	8/16	4/16

The LAMP-CRISPR/Cas12b platform showed exceptional specificity, producing positive signals exclusively for GBS among 11 tested clinically relevant bacterial strains (Fig. S2).

### *Cis*-cleavage and *trans*-cleavage activity of AapCas12b under different temperatures

Given the distinct temperature requirements for LAMP (60°C) and RPA (39°C) amplification, and considering the known temperature sensitivity of AapCas12b, we hypothesized that the suboptimal performance of the RPA-CRISPR/Cas12b system might be attributed to temperature effects. We therefore systematically evaluated the temperature dependence of both *cis*-cleavage and *trans*-cleavage activities of AapCas12b, as measured by the Δ*Rn* values representing fluorescence signal increments.

**Fig 3:**
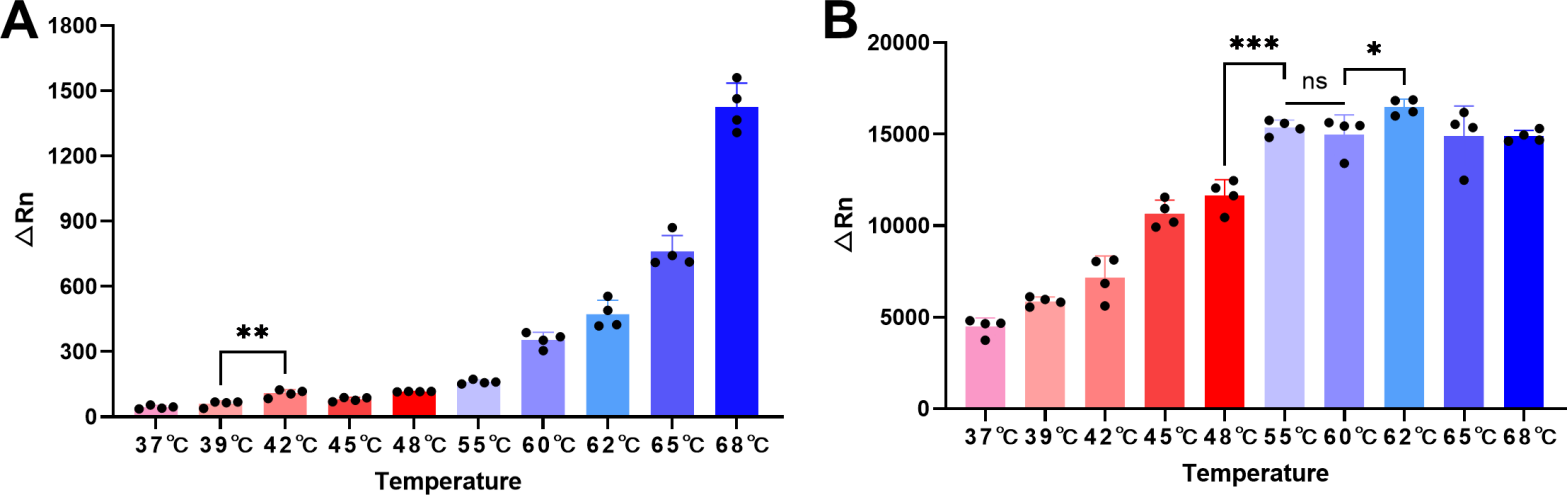
Temperature profiles of AapCas12b (**A**) *cis*-cleavage activity (dsDNA targets) and (**B**) *trans*-cleavage activity (ssDNA reporters). Reactions were performed across a 37–68°C gradient. Statistical significance is indicated as: *P < 0.05, **P < 0.01, and ***P < 0.001; ns: no significance.

It showed a clear increasing trend of *cis*-cleavage activity of AapCas12b along with the temperature build-up, reaching maximum activity at 68°C (Fig. 3A). The Δ*Rn* value generated at 42°C was significantly enhanced when compared to that at 39°C (*P* = 0.0052), suggesting an apparent activation of the *cis*-cleavage activity of AapCas12b under 42°C. Figure 3B illustrated the results of *trans*-cleavage activity, which peaked at 62°C, with significantly higher activity than at 60°C (*P* = 0.0407). Consequently, 39°C was selected to minimize the *cis*-cleavage activity, while 62°C was set as the optimal temperature to max the *trans*-cleavage activity of AapCas12b.

### Two-temperature strategy and low-concentration template enhance detection sensitivity

**Fig 4:**
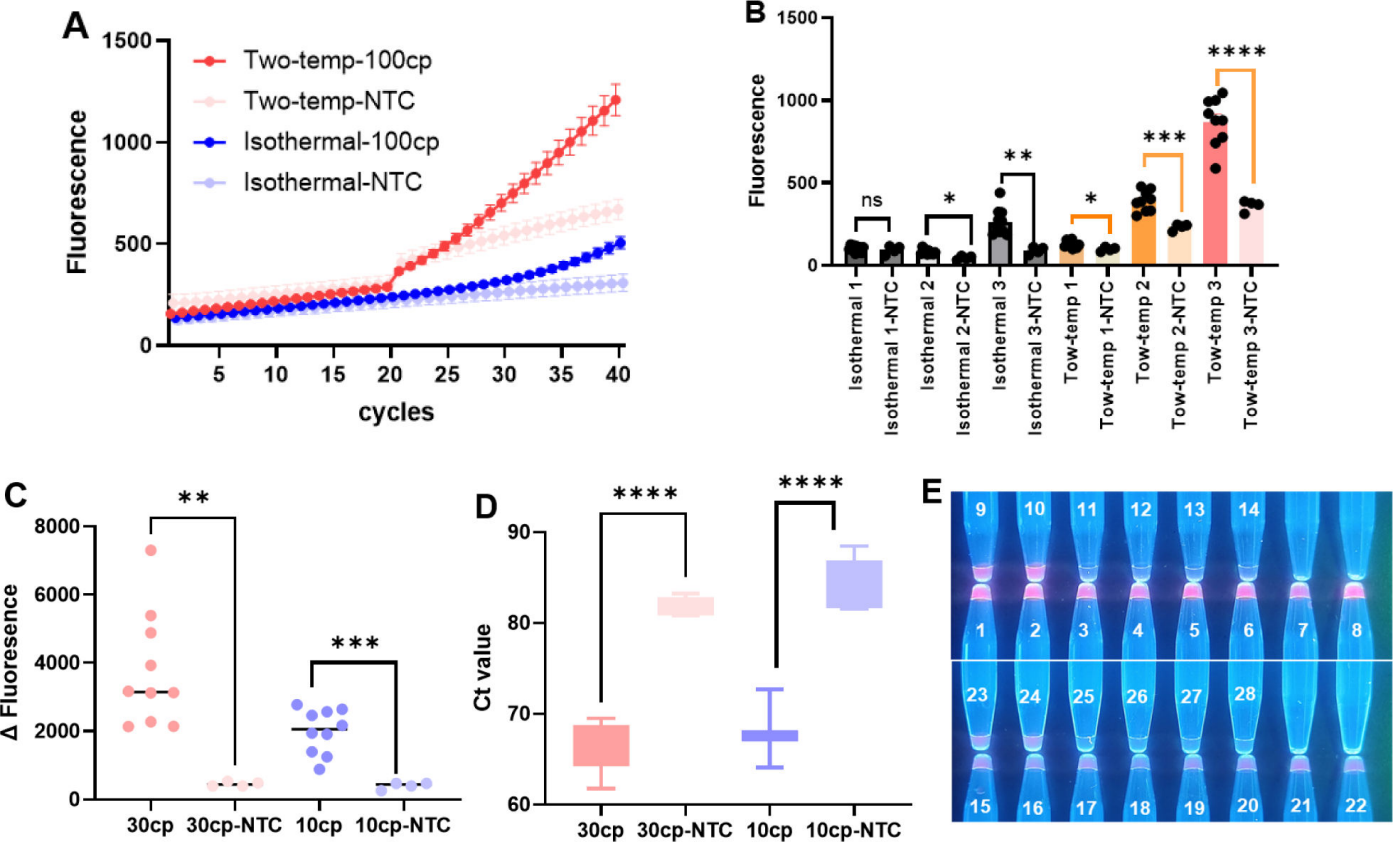
Optimization of the one-pot two-temperature RPA-CRISPR/Cas12b system for enhanced sensitivity. (**A**) Real-time fluorescence curves comparing isothermal (39°C) and two-temperature (two-temp, 39°C→62°C transition at cycle 20) reactions using 100 copies/test template. (**B**) Endpoint fluorescence analysis of different reaction conditions: isothermal reactions (39°C for 20/30/40 cycles) versus two-temperature protocols (transition to 62°C at cycles 20/30/40). “Isothermal 1, 2, and 3” indicate constant 39°C reactions for 20, 30, and 40 cycles, respectively. “Two-temp 1, 2, and 3” refer to two-step reactions with a temperature shift to 62°C at cycles 20, 30, and 40, respectively. (**C**) Fluorescence signal enhancement (Δ Fluorescence) for low-copy samples (30 and 10 copies/test) versus NTC. (**D**) Comparative Ct values demonstrating significant discrimination between low-copy samples and NTC. (**E**) Visual detection under UV/blue light showing clear differentiation between positive samples (30 copies: wells 1–10; 10 copies: wells 15–24) and NTC (wells 11–14 and 25–28). All two-step reactions comprised 39°C for 40 min followed by 62°C for 5 min. Statistical significance is indicated as: *P < 0.05, **P < 0.01, ***P < 0.001, and ****P < 0.0001; ns, no significance; NTC, no-template control.

To verify the superiority of this one-pot two-temperature strategy, we conducted fluorescence kinetics tests of the RPA-CRISPR/Cas12b system, respectively, with distinct isothermal protocols (39°C for 45 min), two-temperature protocols (39°C for 40 min→ 62°C for 5 min), and extended reactions (up to 40 min) for both conditions. Fluorescence curves demonstrate a steeper fluorescence signal rise in the two-temp group (red lines) compared to the isothermal group (blue lines) (Fig. 4A). Figure 4B revealed that the two-temperature group exhibited a significant boost in signal after switching to 62°C, particularly when the shift occurred at cycle 20 (Two-temp 3), where the endpoint fluorescence is highest, with a significant difference between NTC. This suggests that the temperature elevation at cycle 20 substantially enhances the cleavage activity of AapCas12b, resulting in stronger signal generation and shorter detection time.

Figure 4C depicted the increase in fluorescence signal (ΔFluorescence) from baseline in performing the two-temperature protocol across different target concentrations. Both 30 and 10 copies/test groups show significantly higher fluorescence than their corresponding NTCs, confirming that the two-temperature protocol improves the sensitivity of detection. Figure 4D manifested that Ct values for 30 and 10 copies/test samples were notably lower than those in the NTC group, indicating reliable detection even at low template concentrations, with faster and more efficient signal accumulation under the two-temperature protocol. Figure 4E displayed the tubes observed under UV blue light revealing distinct fluorescence bands in samples with 30 and 10 copies/test, while the NTC group showed no visible signal. These results demonstrate that the two-temperature protocol enables sensitive detection without the need for specialized equipment, supporting its applicability in field or POC settings.

### The establishment of extraction-free methods for GBS detection

To enable true sample-to-answer detection, we optimized nucleic acid release methods bypassing conventional extraction. While viral lysis methods (e.g., HUDSON) exist for CRISPR compatibility, gram-positive GBS requires more rigorous lysis (37). We evaluated three buffers and processing methods.

As depicted in Fig. S3A, among the three tested buffers, 1× RLB15 produced the lowest Ct values (~16), followed by 1× DirectStore (~18) and BL01 (~21). The differences between RLB15 and the other two buffers were statistically significant, indicating that RLB15 enables earlier detection and higher amplification efficiency. As shown in Fig. S3B, the fluorescence output followed the same trend, with 1× RLB15 achieving the highest signal (~1,100), significantly exceeding BL01 (~500) and DirectStore (~700). These results confirm RLB15’s superior compatibility with the CRISPR-based detection system.

Figure S3C shows that heat treatment at 95°C for 5 min significantly improved detection (with reduced Ct value) versus room temperature lysis, and room temperature lysis performed comparably to conventional kit extraction (ns), suggesting that a simple heating step or room temperature lysis directly is feasible to replace extraction protocols without compromising sensitivity.

### Clinical validation of the assay for GBS detection

**Fig 5:**
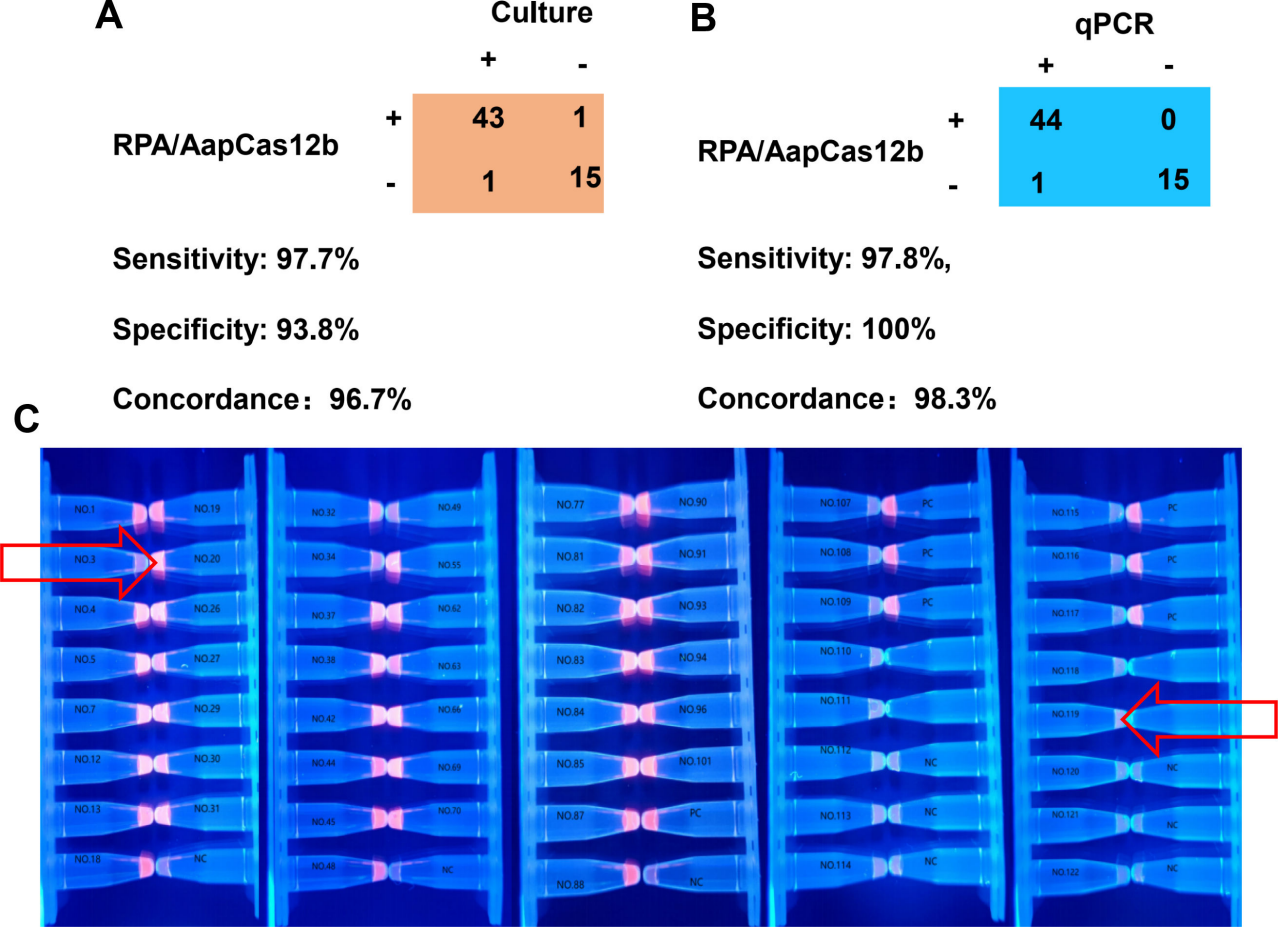
Clinical validation of the extraction-free one-pot two-temperature RPA-CRISPR/Cas12b assay. (**A**) Comparison between CRISPR results and culture-based detection in 60 clinical samples. (**B**) Comparison between CRISPR results and a commercial qPCR kit. (**C**) Visual fluorescence results of clinical samples under UV light. Two samples indicated by red arrows represent discrepant cases: the left sample was culture-positive but CRISPR-negative, while the right sample was culture-negative but CRISPR-positive.

We conducted a performance evaluation using 60 clinical vaginal-rectal swab samples to validate our extraction-free, two-temperature RPA-CRISPR/Cas12b detection system. Compared to traditional culture, the assay achieved a sensitivity of 97.7%, specificity of 93.8%, and an overall agreement of 96.7% (Fig. 5A). When benchmarked against the qPCR method, sensitivity and specificity improved slightly to 97.8% and 100%, respectively, with a 98.3% overall concordance (Fig. 5B). Figure 5C presents a visual fluorescence readout that showed clear discrimination between positive and negative samples. Two discrepant cases identified (pointed out by arrows): Culture+/CRISPR− sample (No. 3): later confirmed as qPCR+ (potentially CRISPR false-negative), and Culture−/CRISPR+ sample (No. 119): Verified as true positive by qPCR.

## DISCUSSION

This study establishes a rapid, extraction-free, one-pot and two-temperature RPA-CRISPR/Cas12b detection system for GBS detection with strong potential for POC potential. Our systematic comparison of LAMP and RPA amplification strategies demonstrates that both can function effectively combined with the CRISPR/Cas12b system, though with distinct performance characteristics. The LAMP-CRISPR/Cas12b system showed superior sensitivity for low-concentration targets, while implementation of a two-temperature protocol significantly improved RPA-CRISPR/Cas12b performance, particularly for low-copy samples. The integration of extraction-free sample processing and visual UV readout further simplifies the workflow, making it particularly suitable for POC testing. Clinical validation revealed excellent agreement with both culture (96.7% concordance) and qPCR (98.3% concordance) reference methods, demonstrating the assay’s reliability for clinical use. Compared to existing CRISPR-based GBS detection methods, our assay outperforms in sample preparation, sensitivity, detection time, and readout simplicity (Table S5).

The selection of optimal primers and sgRNAs represents a critical factor in CRISPR-based detection systems. While RPA offers advantages in primer requirements and cost-effectiveness (38). The performance of CRISPR/Cas relies on well-designed sgRNA, which contains a scaffold (formed by several stem-loop) for Cas protein binding and a specific sequence complementary to target DNA sequence (39, 40). Our data demonstrate that sgRNA7 consistently performs best in both systems, appears particularly well-suited for AapCas12b-mediated detection; hence, sgRNA7 is selected as the optimal candidate for the following assays.

Extensive comparisons have been conducted to verify the performance of RPA and LAMP coupling with CRISPR detection platforms. Our comparative analysis builds upon previous reports describing RPA’s stronger terminal signals but slower kinetics (41), versus LAMP’s more stable amplification profile (42). The superior performance of LAMP-CRISPR/Cas12b, particularly for low-copy targets, highlights its advantages when sensitivity is paramount, such as in early infection screening. However, the precise mechanisms underlying these differences warrant further investigation.

According to the standard specification of commercial kits, we conducted the RPA-CRISPR/Cas12b assay under 39°C. Considering that AapCas12b shows cleavage activity at near 60°C (43), we hypothesized that temperature might be the key factor affecting the sensitivity of the RPA-CRISPR/Cas12b reaction. By performing this system under a large temperature range, we manifested that 39°C is the most preferred temperature at which AapCas12b shows a minimized *cis*-cleavage activity, while 62°C is the optimal temperature to fully exert its *trans*-cleavage activity. These findings suggest that AapCas12b maintains high *trans*-cleavage efficiency even at high temperature ranges, making it well-suited for thermostable detection platforms. Due to the relatively low temperature suitable for RPA assay (37–42°C), this result provides a clue for proposing a one-pot two-temperature strategy.

By initiating amplification at a lower temperature (39°C) suitable for RPA and then shifting to a higher temperature (62°C) beneficial to AapCas12b *trans*-cleavage activity, we improved the sensitivity of RPA-CRISPR/Cas12b device for GBS detection. This approach takes advantage of both efficient isothermal amplification and robust CRISPR-based signal generation, particularly available for low-copy or borderline samples in clinical diagnostics (44, 45). At the same time, the strategy addresses one of the key limitations in RPA-CRISPR assays, where signal strength can be inconsistent at lower temperatures due to suboptimal Cas enzyme activity (46, 47). By temporally decoupling amplification and cleavage conditions, the two-temp approach effectively leverages the strengths of both RPA and AapCas12b, offering a practical and efficient solution for rapid molecular diagnostics.

The challenge of balancing ease of use and diagnostic accuracy in CRISPR platforms hampers its wide application in clinical settings (48, 49). To validate the performance of the extraction-free, one-pot, and two-temperature RPA-CRISPR/Cas12b system in clinical samples, we employed 60 vaginal-rectal swab specimens for GBS detection. It achieved a sensitivity of 97.7% and a specificity of 93.8% when compared to the conventional culture method, with an overall accordance of 96.7%. The overall concordance between our RPA-CRISPR/Cas12b platform and PCR was 98.3%. The assay detected one additional positive case (Fig. 5C, No. 119) missed by culture, suggesting that the CRISPR assay may detect certain low-abundance sample or sample that culture method might miss. This result underscores the assay’s high sensitivity and reliability in clinical practice. However, the CRISPR assay also reported a false negative sample (Fig. 5C, No. 3), which may be attributed to some potential interfering substance in this sample. The performance of the RPA-CRISPR/Cas12b system should be validated in larger sample sizes. In future developments, incorporating a reference card could significantly enhance end-user convenience during visual interpretation of results. In addition to this, the integration of extraction-free processing and visual readout further simplifies the experiment, data processing, and interpretation procedures, aligning with the needs for POC diagnostics, especially in low-resource environments.

### Conclusions

Taken together, we have developed an integrated RPA-CRISPR/Cas12b platform that combines analytical sensitivity, operational simplicity, and visual readout for GBS detection. This system addresses key requirements for decentralized screening in resource-limited settings and urgent clinical scenarios. The modular design principle could be readily adapted for detecting other pathogens in future POC applications. Current limitations include dependence on controlled heating equipment and the need for larger-scale clinical validation. Future work should explore multiplexing capabilities and integration with portable devices to further enhance the system’s utility in diverse field settings.

## AFTER EPUB

*[This article was published on 19 September 2025 with an error in the title. The title was corrected in the current version, posted on 26 September 2025.]*

## SUPPLEMENTAL MATERIAL

Supplemental material jcm.00819-25-s0001.docx Graphical abstract; Figures S1 to S3; Tables S1 to S5.

ASM does not own the copyrights to Supplemental Material that may be linked to, or accessed through, an article. The authors have granted ASM a non-exclusive, world-wide license to publish the Supplemental Material files. Please contact the corresponding author directly for reuse.
