# Supplementary material for: An extraction-free and one-pot two-temperature CRISPR/Cas12b system for visual detection of Group B Streptococcus by integrating with RPA
Source: J Clin Microbiol. 2025 Sep 19;63(10):e00819-25. doi: 10.1128/jcm.00819-25 (PMC12506078; doi:10.1128/jcm.00819-25)
Supplement: Supplemental material — Graphical abstract; Figures S1 to S3; Tables S1 to S5. [file jcm.00819-25-s0001.docx]

**An extraction-free and one-pot two-temperature CRISPR/Cas12b system for visual detection of group B *Streptococcus* by integrating with PRA**


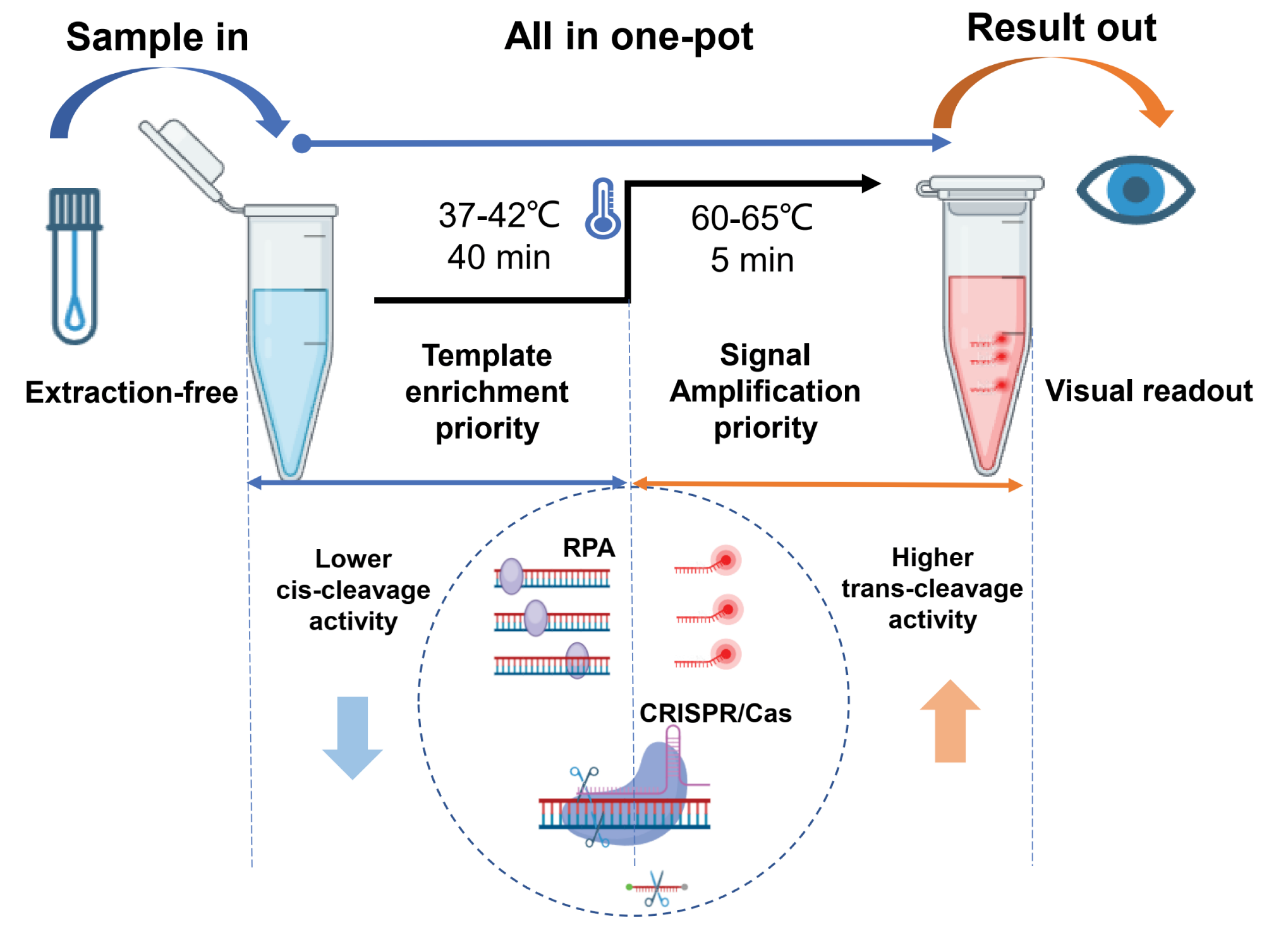


**Graphical abstract**


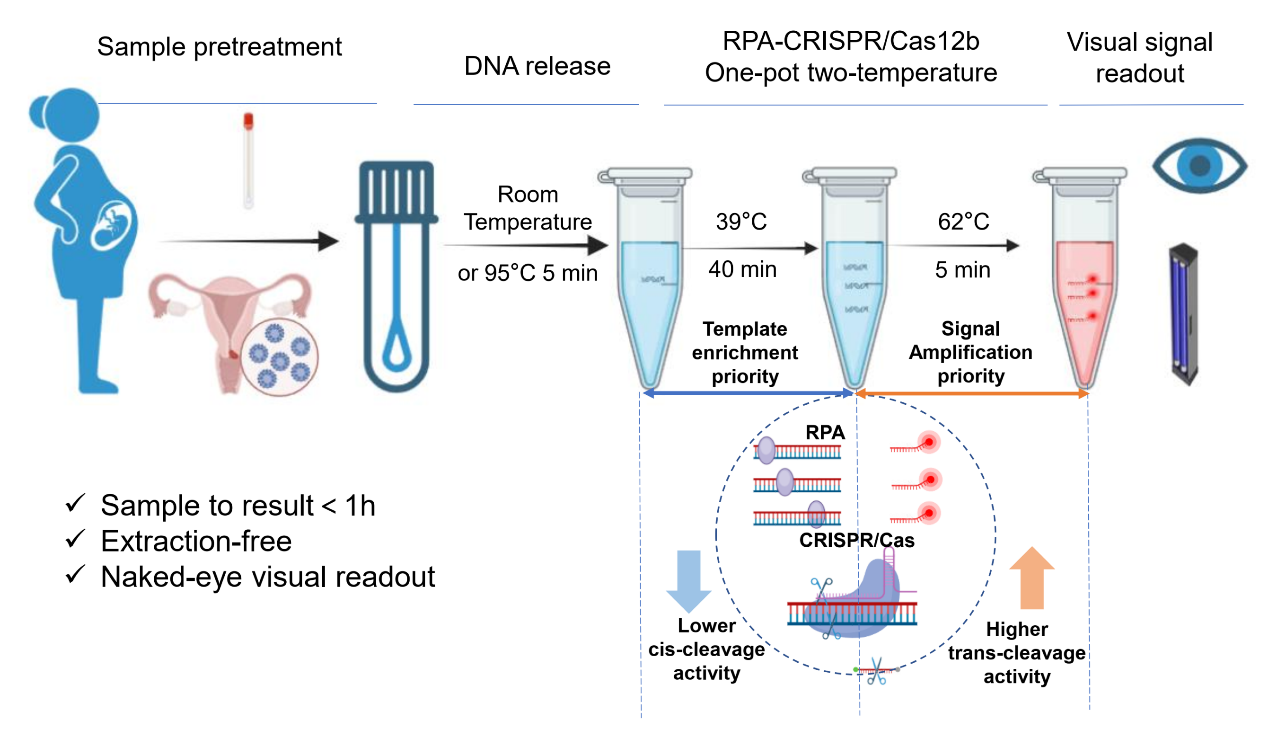


**Scheme 1** Workflow of the extraction-free, one-pot two-temperature RPA-CRISPR/Cas12b visual detection system for GBS. This schematic outlines the simplified workflow of our integrated GBS detection platform, which integrates sample processing, isothermal amplification, and CRISPR-based detection in a single-tube format. The system eliminates the need for nucleic acid extraction and specialized instrumentation, offering a practical solution for point-of-care testing.


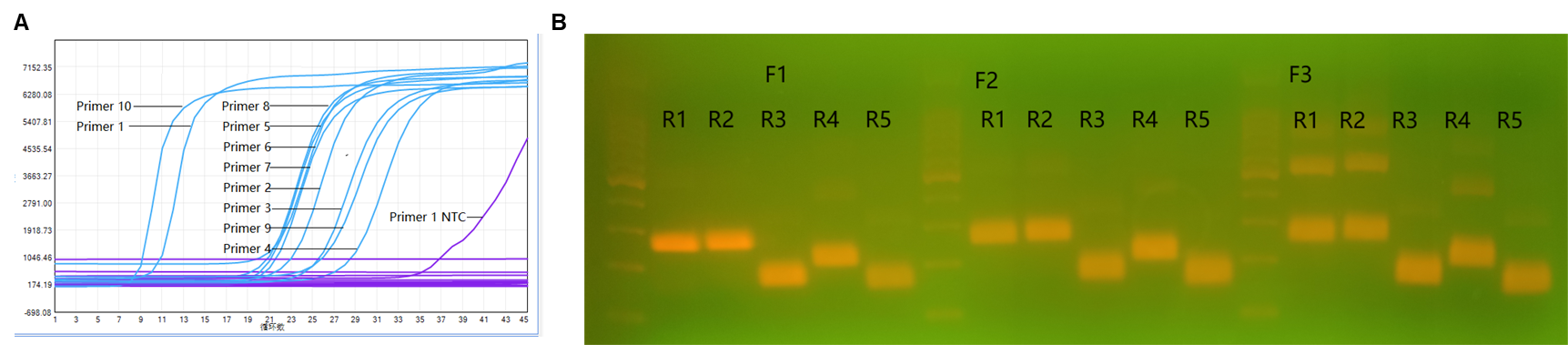


**Figure S1** The primer selection for LAMP (A) and RPA (B).


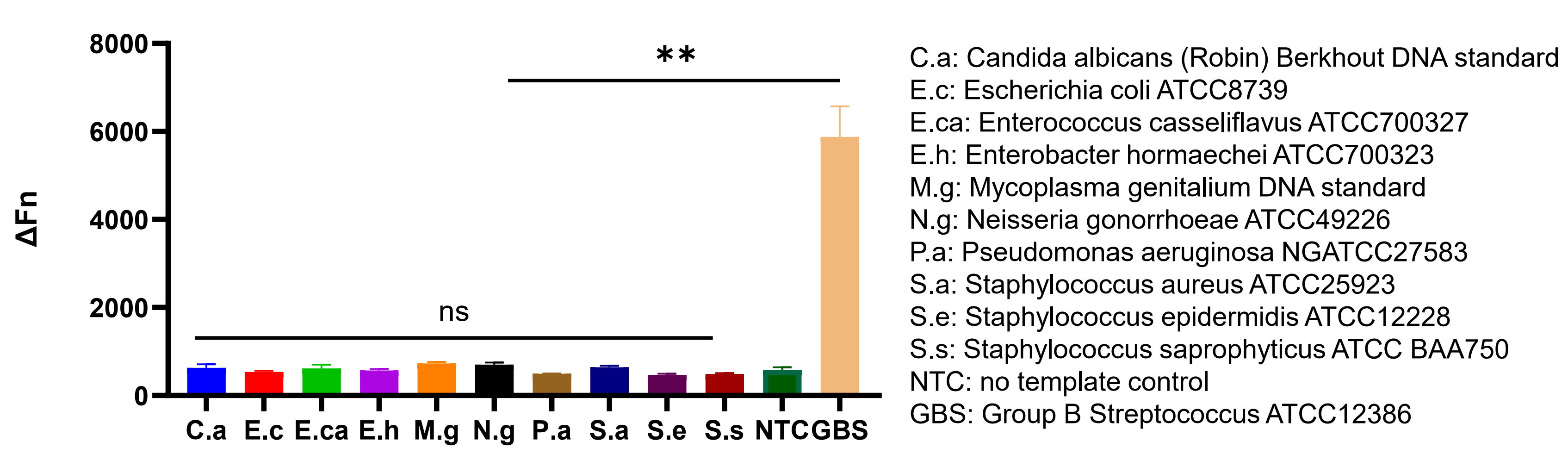


**Figure S2** Specificity analysis of the PRA-CRISPR/Cas12b assay for GBS detection.


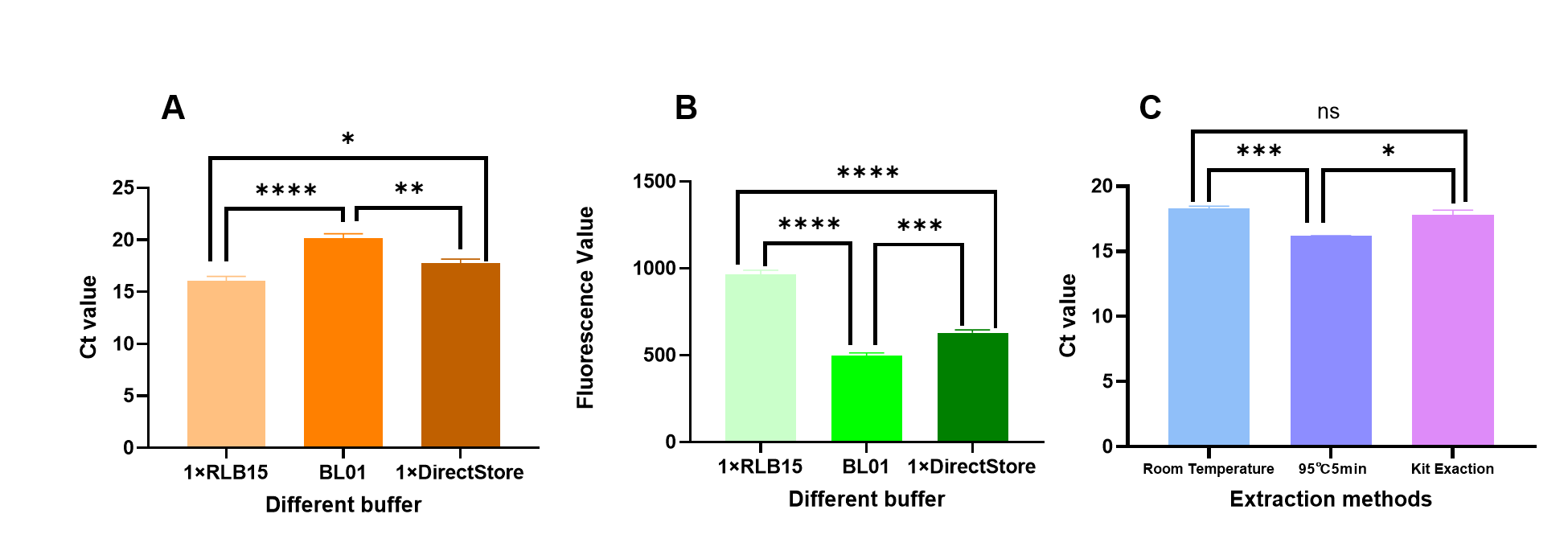


**Figure S3** Evaluation of different buffer types and extraction-free sample preparation methods for LAMP-CRISPR/Cas12b detection of GBS. Comparison of Ct values (A) and endpoint fluorescence (B) for three lysis buffers: 1×RLB15, BL01, and 1×DirectStore. The 1×RLB15 buffer yielded significantly lower Ct values and higher fluorescence intensity than the other two buffers, indicating better compatibility with the one-pot detection system. (C) Comparison of extraction-free treatment methods. Heating at 95 °C for 5 min significantly improved sensitivity compared to room temperature treatment (***p < 0.001). No significant difference was observed between the 95 °C treatment group and the standard kit-based extraction group, suggesting that the heat-based extraction-free protocol provides comparable performance. Statistical significance is indicated as *p < 0.05, **p < 0.01, ***p < 0.001, ****p < 0.0001, and ns: no significance.

**Table S1** The information of LAMP primers.

| LAMP Primers | Sequence(5’ to 3’) | Production size (bp) |
| --- | --- | --- |
| GBS-cfb-F3-1 | AGCAATCACTTTTTCAACTCAA | 206 |
| GBS-cfb-B3-1 | GGTAGCTCTATCAGTTGGTT |  |
| GBS-cfb-F1P-1 | GCGAATAACTAGCTTAGTTATCCC  ACATTTAGCAAATAAGGTTAGTCAAG |  |
| GBS-cfb-B1P-1 | AGATCCATTTGCTTCAGTTGATTCAC  AGGATAAGTTAAAACCTTTTGTTC |  |
| GBS-cfb-LB-1 | AGCTCAAGTTAACGATGTAAAGGCA |  |
| GBS-cfb-F3-2 | AGCTGTATTAGAAGTACATGCT | 214 |
| GBS-cfb-B3-2 | GGCACGCAATGAAGTCTT |  |
| GBS-cfb-F1P-2 | CCATTTGCTGGGCTTGATTATTAC GATCAAGTGACAACTCCACA |  |
| GBS-cfb-B1P-2 | TCAAAGATAATGTTCAGGGAACAGA TTCAACACTAGTAATAGCCTCAT |  |
| GBS-cfb-F3-3 | GGTGCATTGTTATTTTCACCA | 234 |
| GBS-cfb-B3-3 | GGCACGCAATGAAGTCTT |  |
| GBS-cfb-F1P-3 | CCATTTGCTGGGCTTGATTATTACT AGTACATGCTGATCAAGTGAC |  |
| GBS-cfb-B1P-3 | TCAAAGATAATGTTCAGGGAACAGA TTCAACACTAGTAATAGCCTCAT |  |
| GBS-cfb-F3-4 | ACTCAACATTTAGCAAATAAGGT | 203 |
| GBS-cfb-B3-4 | GATTTTGTATAGATGGTAGCTCTA |  |
| GBS-cfb-F1P-4 | CTGAAGCAAATGGATCTAAAATGCG TAGTCAAGCAAATATTGATATGGGA |  |
| GBS-cfb-B1P-4 | AAGCTCAAGTTAACGATGTAAAGGC TCAGTTGGTTTTAAATCAGGAT |  |
| GBS-cfb-F3-5 | AGAAGCCTTAACAGATGTGA | 203 |
| GBS-cfb-B3-5 | CAGGATAAGTTAAAACCTTTTGTTC |  |
| GBS-cfb-F1P-5 | CTAGCTTAGTTATCCCAAATCCCAT GAAGCAATCACTTTTTCAACTC |  |
| GBS-cfb-B1P-5 | ATTCGCATTTTAGATCCATTTGCTT GCCTTTACATCGTTAACTTGAG |  |
| GBS-cfb-F3-6 | AAGACTTCATTGCGTGCC | 235 |
| GBS-cfb-B3-6 | CCTTTACATCGTTAACTTGAGC |  |
| GBS-cfb-F1P-6 | TGAGTTGAAAAAGTGATTGCTTCAA ATGATTTGAATTCTATTGGTAGTCG |  |
| GBS-cfb-B1P-6 | ATGGGATTTGGGATAACTAAGCTAG ATTGAATCAACTGAAGCAAATGG |  |
| GBS-cfb-F3-7 | AAGCCTTAACAGATGTGATTG | 227 |
| GBS-cfb-B3-7 | GGTAGCTCTATCAGTTGGTT |  |
| GBS-cfb-F1P-7 | CTAGCTTAGTTATCCCAAATCCCAT CTTTTTCAACTCAACATTTAGCA |  |
| GBS-cfb-B1P-7 | ATTCGCATTTTAGATCCATTTGCTT AACCTTTTGTTCTAATGCCTT |  |
| GBS-cfb-F3-8 | CAAATAAGGTTAGTCAAGCAAAT | 190 |
| GBS-cfb-B3-8 | GATTTTGTATAGATGGTAGCTCTA |  |
| GBS-cfb-F1P-8 | AATTGAATCAACTGAAGCAAATGGA TATGGGATTTGGGATAACTAAGC |  |
| GBS-cfb-B1P-8 | AAGCTCAAGTTAACGATGTAAAGGC TCAGTTGGTTTTAAATCAGGAT |  |
| GBS-cfb-F3-9 | CATTTAGCAAATAAGGTTAGTCAAG | 199 |
| GBS-cfb-B3-9 | GATTTTGTATAGATGGTAGCTCTA |  |
| GBS-cfb-F1P-9 | TGAAGCAAATGGATCTAAAATGCGA CAAATATTGATATGGGATTTGGGA |  |
| GBS-cfb-B1P-9 | AAGCTCAAGTTAACGATGTAAAGGC TCAGTTGGTTTTAAATCAGGAT |  |
| cfb4-F3-10 | AGAAGCCTTAACAGATGTGA | 219 |
| cfb4-B3-10 | TCAGTTGGTTTTAAATCAGGA |  |
| cfb4-FIP-10 | TCCCAAATCCCATATCAATATTTGCAG  CAATCACTTTTTCAACTCA |  |
| cfb4-BIP-10 | ATTCGCATTTTAGATCCATTTGCTACYT  TTTGTTCTAATGCCTT |  |
| cfb4-LF-10 | TGACTAACCTTATTTGYTAAATG |  |
| cfb4-LB-10 | ATTAAAGCTCAAGTTAACGATG |  |

**Table S2** Sequences of RPA primers.

| PRA pimer | Sequence(5’ to 3’) |
| --- | --- |
| GBS-RPA-F1 | gattgaagcaatcactttttcaactcaaca |
| GBS-RPA-F2 | tgaagcaatcactttttcaactcaacattt |
| GBS-RPA-F3 | agcaatcactttttcaactcaacatttagc |
| GBS-RPA-R1 | gcgtgtattccagatttccttatcaagttt |
| GBS-RPA-R2 | gtaaagcgtgtattccagatttccttatca |
| GBS-RPA-R3 | accttttgttctaatgcctttacatcgtta |
| GBS-RPA-R4 | ggtagctctatcagttggttttaaatcagg |
| GBS-RPA-R5 | tgcctttacatcgttaacttgagctttaat |

**Table S3** Sequences of sgRNAs of Cas12b.

| sgRNA No. | Sequence(5’-3’) |
| --- | --- |
| cfb-10-sgRNA1 | GUCUAGAGGACAGAAUUUUUCAACGGGUGUGCCAAUGGCCACUUUCCAGGUGGCAAAGCCCGUUGAGCUUCUCAAAUCUGAGAAGUGGCACGGAUAACUAAGCUAGUUAUU |
| cfb-10-sgRNA2 | GUCUAGAGGACAGAAUUUUUCAACGGGUGUGCCAAUGGCCACUUUCCAGGUGGCAAAGCCCGUUGAGCUUCUCAAAUCUGAGAAGUGGCACCUUCAGUUGAUUCAAUUAAA |
| cfb-10-sgRNA3 | GUCUAGAGGACAGAAUUUUUCAACGGGUGUGCCAAUGGCCACUUUCCAGGUGGCAAAGCCCGUUGAGCUUCUCAAAUCUGAGAAGUGGCACAAUCAGGAUAAGUUAAAACC |
| cfb-10-sgRNA4 | GUCUAGAGGACAGAAUUUUUCAACGGGUGUGCCAAUGGCCACUUUCCAGGUGGCAAAGCCCGUUGAGCUUCUCAAAUCUGAGAAGUGGCACUAAUGCCUUUACAUCGUUAA |
| cfb-10-sgRNA5 | GUCUAGAGGACAGAAUUUUUCAACGGGUGUGCCAAUGGCCACUUUCCAGGUGGCAAAGCCCGUUGAGCUUCUCAAAUCUGAGAAGUGGCACAGCUUUAAUUGAAUCAACUG |
| cfb-10-sgRNA6 | GUCUAGAGGACAGAAUUUUUCAACGGGUGUGCCAAUGGCCACUUUCCAGGUGGCAAAGCCCGUUGAGCUUCUCAAAUCUGAGAAGUGGCACAUUGAAUCAACUGAAGCAAA |
| cfb-10-sgRNA7 | GUCUAGAGGACAGAAUUUUUCAACGGGUGUGCCAAUGGCCACUUUCCAGGUGGCAAAGCCCGUUGAGCUUCUCAAAUCUGAGAAGUGGCACAAUCAACUGAAGCAAAUGGA |
| cfb-10-sgRNA8 | GUCUAGAGGACAGAAUUUUUCAACGGGUGUGCCAAUGGCCACUUUCCAGGUGGCAAAGCCCGUUGAGCUUCUCAAAUCUGAGAAGUGGCACAAGCUCAAGUUAACGAUGUA |

**Table S4** Sequences of dsDNA probes.

| **Probe** | **Sequence(5’ to 3’)** |
| --- | --- |
| cfb-sg7-dsDNA-Probe-R | attttagatccatttgcttcagttgattcaattaaagctcaagttaacga |
| cfb-sg7-dsDNA-Probe-F | tcgttaacttgagctttaattgaatcaactgaagcaaatggatctaaaat |

**Table S5** Comparison of our developed assay with the existing GBS detection assay

| **No.** | **Extraction** | **Amplification (target)** | **CRISPR/Cas** | **Detection system** | **Signal readout** | **Sensitivity** | **Time** | **Comments** | **Ref.** |
| --- | --- | --- | --- | --- | --- | --- | --- | --- | --- |
| 1 | 25min  (99℃ 10 min) | RPA (atoB)  37℃ 30 min | Cas13  37℃ 20 min | two-pot | ABI 7500 | 60 CFU/ml  5 copies/reaction | ＞1 h | The operation is cumbersome, prone to aerosol contamination, and higher cost. | Jiang,2021 |
| 2 | 100℃10 min | RPA (cfb)  37℃ 15 min | LbaCas12a  37℃ 10 min | two-pot | Quant Studio 3 | 5 copies μL^−1^ | 35 min | The operation is cumbersome and prone to aerosol contamination. | Yu,2023 |
| 3 | 100℃ 5 min | RPA (cfb) | AsCas12a  39℃ 50 min | one-pot | CFX96  LFA assay | 16.6 aM | 55 min | The ssDNA-modified crRNA is cumbersome and higher cost. | Zeng,2023  Ma,2025 |
| 4 | Extraction-free release directly | MIRA (cAMP) | LbCas12a  39℃ 30 min | one-pot | UV light  LFA assay | 32 copies/reaction  (0.64copies/μL) | 30 min | The suboptimal PAM is not universal and its selection is random. | Zheng,2024  Tong,2024  Chen,2025 |
| 5 | Extraction-free release directly or 95℃ 5 min | RPA (cfb) | AapCas12b  39℃ 40 min  62℃ 5min | one-pot | UV light | 10 copies/reaction  (1 copy/μL) | 45 min | The two-temperature strategy is universal and simply operation. | This work |
